# Effect of Seasonality and Ecological Factors on the Prevalence of the Four Malaria Parasite Species in Northern Mali

**DOI:** 10.1155/2012/367160

**Published:** 2012-03-14

**Authors:** Ousmane A. Koita, Lansana Sangaré, Hammadoun A. Sango, Sounkalo Dao, Naffet Keita, Moussa Maiga, Mamadou Mounkoro, Zoumana Fané, Abderrhamane S. Maiga, Klénon Traoré, Amadou Diallo, D. J. Krogstad

**Affiliations:** ^1^Laboratoire de Biologie Moléculaire Appliquée, Faculté des Sciences et Techniques, Université de Bamako, BP 2191, Bamako, Mali; ^2^Département d'Epidémiologie et de Santé Publique, Faculté de Médecine, Pharmacie et d'OdontoStomatologie, Université de Bamako, BP 1805, Bamako, Mali; ^3^Service des Maladies Infectieuses, Hôpital National du Point G, BP 1805, Bamako, Mali; ^4^Direction Régionale de la Santé de Gao, Ministère de la Santé, BP 232, Gao, Mali; ^5^Faculté des Langues des Arts et des Sciences Humaines, Université de Bamako, BP E 3637, Bamako, Mali; ^6^Institut National de Recherche en Santé Publique, BP 1771, Bamako, Mali; ^7^Department of Tropical Medicine, School of Public Health and Tropical Medicine, Tulane University, New Orleans, LA 70112, USA

## Abstract

*Background*. We performed 2 cross-sectional studies in Ménaka in the Northeastern Mali across 9 sites in different ecological settings: 4 sites have permanent ponds, 4 without ponds, and one (City of Ménaka) has a semipermanent pond. We enrolled 1328 subjects in May 2004 (hot dry season) and 1422 in February 2005 (cold dry season) after the rainy season. *Objective.* To examine the seasonality of malaria parasite prevalence in this dry northern part of Mali at the edge of the Sahara desert. *Results*. Slide prevalence was lower in hot dry than cold dry season (4.94 versus 6.85%, *P* = 0.025). Gametocyte rate increased to 0.91% in February. Four species were identified. *Plasmodium falciparum* was most prevalent (74.13 and 63.72%). *P. malariae* increased from 9.38% to 22.54% in February. In contrast, prevalence of *P. vivax* was higher (10.31%) without seasonal variation. Smear positivity was associated with splenomegaly (*P* = 0.007). Malaria remained stable in the villages with ponds (*P* = 0.221); in contrast, prevalence varied between the 2 seasons in the villages without ponds (*P* = 0.004). *Conclusion*. Malaria was mesoendemic; 4 species circulates with a seasonal fluctuation for *Plasmodium falciparum*.

## 1. Introduction

Malaria is still an overwhelming public health problem in the sub-Saharan Africa, more than 90% of the disease burden occurs in Africa [1]. Children under 5 years of age and pregnant women constitute the major groups for developing life-threatening situation in malaria [2]. Case fatality rates for cerebral malaria and severe anemia among children have been estimated, respectively, 19 and 13% [3]. Acquisition of immunity against malaria infection requires a long exposure to infective bite of anopheline mosquitoes [4] and the number of those infective bites depends on several factors such as human host, parasite genetic make-up, and ecology [5].

Thus, distribution of malaria relies on ecological factors such as rainfall in the Sahel; anopheline mosquito, the vector of malaria, responsible for transmitting the parasite from one host to another needs enough water to breed. Based on previous studies [6, 7], it has been observed that prevalence of malaria infection ranges from 70–85% in the south of Mali with 1,000 mm of annual rain to 0.5% in the north [8] where the annual rainfall is below 400 mm. Sahara constitutes a buffer zone between the southern part of Africa and the Mediterranean border. In contrast to the wet southern part, the belief is that malaria does not exist in the Sahara or can be epidemic [9]. However, bodies of water like ponds are scattered through this region and constitute settlements for nomadic people. Malaria epidemic may occur in such establishment among susceptible population with housing easily accessible to mosquitoes [10]. Strategies to control malaria must be based on epidemiological entities; therefore all parameters should be assessed in each distinct ecological zone to rationalize the use of scarce resources. Several studies were carried out in southern part of Mali which provide prevalences of infection during the rainy and dry seasons, varying from 85% to 55%, respectively [6, 7], and only 3 species of malaria parasites have been described. *P. malariae* represents only 2 to 3% of infected patients and no infection with *P. vivax* was observed in dark skin subjects during all parasitological investigations carried out so far in Mali. The northern Mali is land of nomadic populations (Tuaregs and Maures) who move around ponds, oueds, and water forages; no epidemiological studies were performed in a prospective manner to identify dynamic of circulating parasites in those areas and to evaluate parasitological parameters of malaria in the North of Mali. Thus, we carried out a cross-sectional study to examine the seasonality of malaria parasite prevalence during dry hot (May 2004) and cold (February 2005) seasons in the District of Ménaka located in the North-Eastern part of Mali.

## 2. Materials and Methods

### 2.1. Study Area

**Figure 1:**
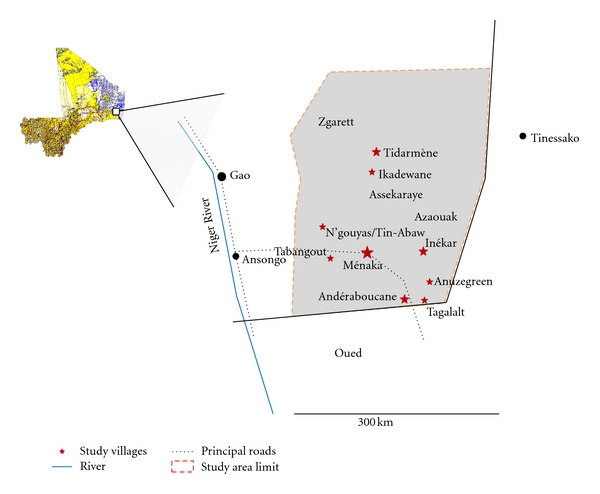
Map of study area. This map shows the 9 sites where the study was performed. Andéramboukane (has a permanent pond) and Talgalat (semipermanent pond, dry at the end of the year) are located near the border with the Republic of Niger (North of Mali). Tin-abaw and and N'gouyass separated by a permanent pound are located between Ménaka and Andéramboukane. Inekar and Anuzegreen that are also located between Menaka and Andéramboukane do not have ponds. Sites such as Tabangout (10 km from Ménaka) and Tidarmane-Ikadewane that are situated in the south of Ménaka, do not have ponds. Ménaka, the capital city, has a semipermanent pond.

The District of Ménaka is located in the northern-East between longitude 1°20 and 4°15 East and latitude 15°20 and 18° north. It is limited by Tinessako (Region of Kidal) in the North, Niger in the East, and by Ansongo in the West (Figure 1). The height above sea level varies between 280 and 350 mm. There are two main seasons (dry and rainy). In dry season, Ménaka is arid with average of temperature of 38°C (varying from 25 to 45°C); it begins from November to May and the rainy season from June to October. Annual rainfall oscillates between 150 mm in the North and 300 mm in the South. This study has been carried out in 9 rural communes and the district of Ménaka. Population of Ménaka is in majority farmers and nomadic breeders [11]. According to the administrative census of 1998, population of Ménaka counts about 73116 inhabitants with a density less than one inhabitant per km^2^. Since 1998, 18% of the populations settled permanently in cities of Ménaka, Andéramboukane, and Inékar. The rest of the population (82%) settled in the nomadic camps around the ponds. The main ethnic group is Kel Tamacheq (nomadic inhabitants), they are about 95%, and the minority groups are seminomadic Sonrhaïs, Haoussas, and Maures. The Daoussahaqs are persons of mixed race of Touaregs and Sonrhaï and speak a dialect close to these two languages. They represent the majority of the active population in sectors of agriculture and cattle breeding [11].

### 2.2. Sample Size and Study Populations

We have carried out a random sampling by relying on subjects who were present at the time of our passage in each site until we reached the estimated number of subjects planned for the study. Based on our stratification, 108 subjects may constitute one unit. A total of 1328 subjects of 12 clusters (108 units per cluster) have been screened for malaria infection in May 2004 in 9 rural and district sites of Ménaka prefecture. We performed 8 months after a second passage in February by including 1422 subjects with 108 units per cluster.

### 2.3. Clinical Examination

Each subject was examined by a physician at teach study site (village). Body axillary's temperature was recorded using an electronic thermometer (ThermoScan, Whyllen) and spleen enlargement was assessed based on Hackett's criteria [12].

### 2.4. Thin and Thick Smears

Thick and thin smears were collected from the fingerstick and then were stained with 3% giemsa (Sigma, St. Louis, MO) in phosphate buffer (pH 7.0) and examined using oil immersion magnification (1000x). Each slide was examined by two microscopists, who estimated the parasite density by counting the number of asexual *P. falciparum* parasites in fields containing 300 white blood cells, and multiplied that number by 25 to estimate the number of asexual *P. falciparum *parasites per *μ*L (based on an average white blood cells count of 7500 per *μ*L—[13]). Slides were considered positive or negative after two readers examined fields containing 300 white blood cells. Slides on which there was disagreement were examined by a third microscopist.

### 2.5. Collection of Filter Paper Blots

Freshly blotted filter papers obtained from each subject were dried in a closed cardboard box to protect them from flies and dust. Each filter paper was then labeled with the subject's study number, placed in an envelope labeled with the same number, and stored at ambient temperature in a file cabinet.

### 2.6. Hematocrit Measurement

A capillary tube (VWR, West Chester, Pa) covered of heparin (anticoagulant) was filled of blood to the feature of calibration. Then, one of the ends was stopped with wax. The blood contained in each capillary tube of each subject was then separated after centrifugation with the Thermo MicroMB centrifuge (Thermo IEC, Needham Heights, MA, USA). Reading was performed according to instructions' of manufacturer using circular microcapillary tube reader (IEC, Needham Heights, MA, USA). In this study, anemia was defined when the hematocrit rate is less than 33 [14].

### 2.7. DNA Amplification Using the Polymerase Chain Reaction (PCR)

*Plasmodium vivax* parasite DNA was extracted from filter paper blots with Chelex-100 method as previously described [15]. Using primers specific for the 5′ and 3′ regions of the MSP-1 for *P. vivax* that flank the repeat region, Nested PCR amplification was performed as described by Alger [16].

### 2.8. Data Analysis

Excel software (Microsoft office 2003) has been used for data entry, the statistical analysis with the SPSS software (Version 11.0 for Window). Pearson chi-square test or Fisher test has been used for prevalence comparison between data obtained in 2004 and 2005. Unpaired *t*-test was performed to compare if there was a difference in terms of the size population between the dry hot season (2004) and dry cold season (2005). The odds ratio test has been estimated to seek the relationship between malaria species and symptoms. A *P* value less than 0.05 was considered as significant.

### 2.9. Ethical Consideration

Protocol was first submitted and approved by the Ministry of Health (Bamako, Mali) and then explained to village's council. After its approval, each participant was then informed about the protocol; care was given to all study participants. Oral informed consent or assent (for children less than 18 years of age) was then obtained from each participant.

## 3. Results

We performed 2 cross-sectional passages, one during the dry season just one month prior to the rainy season which begins between June and July. The second survey was conducted in February 3 months after the rainy season ending in October. We could not perform the study during the rainy season because most of children enrolled in the study (May 2004 and February 2005) were in the school summer break which occurs during the malaria transmission season (June-September) and were not accessible. However, data collected during the dry and cold periods allowed us to examine the fluctuation of parasitological parameters among populations in the 9 sites of Ménaka. There was not a significant difference between our study populations during the 2 passages (Table 1). Thus, 1328 subjects were included in May 2004 versus 1422 in February 2005. No statistical variation was observed among populations of the 9 sites during the 2 passages (Table 1). In addition, subjects between 0 and 9 years of age were comparable during the 2 passages (May 2004 and February 2005) with a *P* value = 0.868 (unpaired *t*-test). Similarly, subjects with age greater than 9 years were also comparable between the 2 passages (May 2004 and February 2005) with *P* value = 0.866 (Table 1).

Comparing the data obtained from the studied populations in 2004 and 2005 (Table 2), there was a significant difference between age groups and the presence of ponds (or absence of ponds), we observed that there were more children in the areas with ponds than areas without ponds (*P* < 0.0001). The same trends were observed with the type of skin (derma); there were more subjects with dark skin in the areas with ponds than areas without ponds during the years 2004 and 2005 (*P* < 0.0001). There were more females in the areas with ponds than areas without ponds in 2004 (*P* = 0.002); in contrast there was no significant difference between gender and presence of (absence) ponds in 2005 (*P* = 0.625).

Parasitological data showed that the 4 species were circulating in Ménaka. Overall prevalence varied from 4.95% in May 2004 (with 64 positive slides in 1293 screened subjects) to 7.06% in February 2005 (with 97 positive smears among 1422 subjects). This seasonal variation in prevalence of malaria infection was statistically significant (Table 3, *P* = 0.0259). However, we could not find any statistically difference between gametocyte prevalence between the 2 periods although the prevalence was higher in cold season with 0.91% (13 out of 1415) versus 0.46% (6 out of 1293) in dry season (Table 3, *P* = 0.2364).

*Plasmodium falciparum* was the most prevalent in dry season (2004) as well as in cold period (2005), but with a higher proportion in dry season 74.13% versus 63.72% in cold season. In 64 infections (2004), 50 were due to *P. falciparum* (Table 3). This proportion decreased during the cold season. Among 97 positive infections with malaria parasites, 56 (63.72%) were caused by *P. falciparum* (Table 3). This variation in terms of *P. falciparum* species prevalence between cold and dry season was statistically significant (Table 3, *P* = 0.03).

Prevalence of *P. malariae* infection increased considerably in cold season. Six infections were due to this species among 64 infected subjects with the 4 species (9.38%) in dry season while among 97 infected persons in cold period, 21 were caused by *P. malariae* (21.64%). There was a significant difference in prevalence of *P. malariae* infection between May 2004 and February 2005 (Table 3, *P* = 0.0419).

*Plasmodium ovale* and *P. vivax* infections represented 3.13% (2/64) and 7.81% (5/64), respectively, in dry season. In cold season, even though the frequency of *P. vivax* was higher 10.31% (10/97), there was not a statistically variation between May 2004 and February 2005 (Table 3, Fisher's exact test *P* > 0.05).

We observed 2 cases of mixed infections, one case of *P. falciparum* and *P. malariae* was identified in dry season (2004) among the 64 positive slides (1.56%), and the number increased to 7 out of 97 positive slides in cold season (2005) with 7.21% (Table 3, Fisher's exact test *P* = 0.147). One case of mixed of 3 species (*P. falciparum, P. ovale*, and *P. vivax*) was identified only in the cold season in 2005 (Table 3).

Prevalence of enlarged spleen was higher during the dry season with 5% (64/1264) than during the cold period with 2.9% (41/1397), and this difference was statistically significant (Table 3, *P* = 0.006).

Clinical symptoms such anemia and fever did not vary significantly between seasons. It was 10.4% (141/1328) and 10.5% and 22.2%, respectively, for anemia and fever (*P* = 0.97 and 0.95 for the 2 symptoms; Table 3).

Relationship between age groups and parasite prevalence was assessed (Table 4); we observed no statistically difference between the 2 passages although the frequency of infection was higher in May among subjects between 0 and 9 years of age with 8.69% (50/575) than in those observed in February with 5.97% (37/619), and *P* value was 0.0753. In contrast, more subjects with age greater than 9 years of age were infected in February than in May, 7.5% (60/796) versus 1.94 (14/718), respectively, and this difference was significant (*P* = 0.000001). Interestingly between the cold (2005) and dry (2004) seasons, children from 0 to 9 years of age and subjects with age greater than 9 years were equally infected; no significant difference was found (Table 4; *P* = 0.0753). In addition, a different picture was observed in May during the dry season; children (0–9 years of age) were frequently infected with 8.69% (50/575) of malaria parasites than older subjects (age > 9 years) with 1.94% (14/718). Difference was statistically significant with *P* = 0.0001 (Table 4). Comparing the plasmodic index within 2005, between 0 and 9 years of age and subjects having more than 9 years of age, the difference was not statistically significant (Table 4, *P* = 0.2890).

We have investigated whether there was a difference in prevalence of parasite infection based on presence of ponds in study's sites. When we compared malaria prevalence in sites with ponds, no statistical difference in May and February (*P* = 0.221) was observed. In contrast, there was a statically difference in the prevalence of malaria between cold and dry season in the villages without ponds (*P* = 0.004). By comparing the prevalence of malaria infection in the villages with ponds and villages without ponds, we did find a difference; the prevalence was higher in sites with ponds than without ponds (Table 5, *P* = 0.0289).

**Figure 2:**
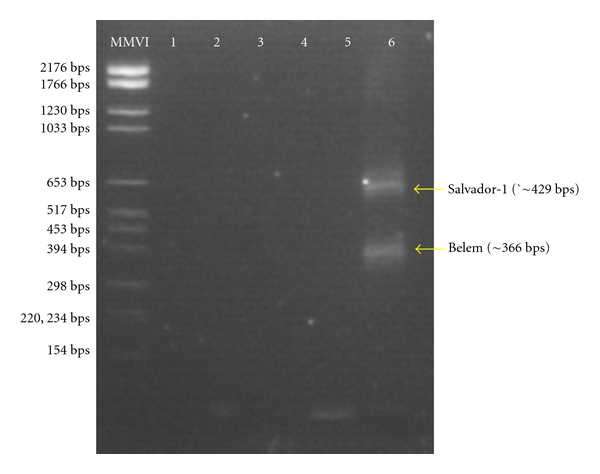
Picture of agarose gel with PCR products. This gel shows a *P. vivax* diagnosis confirmation by nested PCR method using primer sequences designed from MSP-5/6 conserved blocks in subject 5 (Andéranboukane 125).

To confirm the presence of *P. vivax* in that part of Mali identified by thick smear, we performed Polymerase Chain Reaction using *P. Vivax* merozoite surface protein-1 block 5 and 6 markers. Belem (366 basepairs) and Salvador-1 (429 basepairs) genotypes have been identified in a single individual, confirming that *P. vivax* was circulating in Mali (Figure 2).

We assessed the relationship between anemia and malaria species (Table 6, OR = 4.633 95% CI [1.826—11.752]), an association between *P*. *malariae* infection and hemoglobin less than 10 g/dL was observed, and prevalence of anemia was 4.6-fold higher in subjects with *P. malariae* infection (33.3%) than in those with other malaria species (9.7%).

## 4. Discussion

We performed 2 cross-sectional studies in Ménaka, which is situated in the Northern part of Mali at the borders with Niger and Algeria. In terms of sample size, the population screened during the passages of May 2004 and February 2005 was comparable (Table 1). However, we observed a different trend of parasite prevalence; prevalence of infection (smear positivity) was lower during the hot dry season than the cold dry season (4.95 versus 7.06%, *P* = 0.003). This fluctuation could be explained by ecological factors such as ponds that contribute to the transmission of malaria parasites beside the rainy season [17–19]. This prevalence suggests that Ménaka must be considered as zone of hypoendemicity for malaria transmission.

Higher prevalence of malaria infection obtained in February 2005 three months after the conclusion of the rainy period may be associated with the presence of ponds in sites with higher prevalence. In addition, prevalence was stable between hot dry period and cold dry season, and no statistically significant variation in parasite prevalence was observed between sites with ponds (*P* = 0.221). In contrast, there was a significant difference in prevalence of malaria infection in the sites without ponds (*P* = 0.004), the prevalence being higher in cold season than during the hot period. Those data taken together will allow one to hypothesize that ponds serve as breeding sites for mosquitoes, which are able to maintain transmission of malaria parasites in absence of rains in the dry part of the country. Guthmann et al. [20] made the relationship between expansion of ponds and increase of malaria incidence. A 50-fold increase of malaria incidence coincided with development of pond surface in the Iquitos region of Peru.

The four species of malaria parasites have been observed during the 2 passages (May 2004 and February 2005); however the proportion of infected people with the four species varied seasonally. *P. falciparum* was most prevalent (74.13 and 63.72%), followed by *P. malariae,* which increased from 9.38 during the dry hot season (May) to 22.54% after the rainy season in February. The high prevalence of *P. malariae* observed in February was unusual; all studies performed in Mali show that the prevalence was always less than 10% [7, 21]. Thus, we suggest that anopheline mosquitoes associated to ponds could be different in transmitting *P. malariae* than those found in South associated mainly with the rainfall. However, entomological studies are needed to identify anopheline species, which breed around the ponds and assess their vectorial capacity. The only data on anopheline mosquitoes were from the north of Mali and obtained between Gao and the border of Algeria; they indicated that *Anopheles gambiae* complex and *Anopheles pharaonsis *were present [8]. Frequency of *P. vivax* and *P. ovale* remained stable over the 2 periods. Interestingly, the prevalence of *P. vivax* was higher with ~10% of infection compared with to the data collected along the Transsaharan road from Bourem to Tessalit at the border with Algeria. In that region, Koita [8] observed that less than 1% of infection is due to *P. vivax*. The stability in the prevalence of *P. vivax* and *P. ovale* could be explained in part by recurrent infection with the release of hypnozoites [22] from the liver on which there is no treatment available in our 9 study sites. Our PCR data confirmed the data obtained by microscopy about the circulation of *P. vivax* at the edge of Sahara desert. Two genotypes Belem (366 basepairs) and Salvador-1 (429 basepairs) have been identified in a single infection. Presence of *P. vivax* parasite in that region was expected, because in that part of Mali live Duffy positive population, who are susceptible to *P. vivax* infection [23]. Our result contrasts with the data obtained in other sub-Saharan Africa countries. Culleton and his colleagues [21] found only 1.4% of *P. vivax* infections in 9 countries in West and Central Africa. This higher prevalence could be explained by the existence of a proportion of Duffy positive subjects in our study population which turns around 40% (Table 2, data not shown).

Although prevalence of anemia was lower compared to that found in South of the country [24], subjects with hemoglobin less than 10 g/dL were infected frequently with *P. malariae.* We observed that children less than 9 years of age were frequently exposed than older subjects during the dry season; however we did not find a difference among our age-groups after the rainy season. This suggests that age is not a factor in preventing *Plasmodium* infection; each subject was at risk for infection at this period of year.

## 5. Conclusion

Four species of malaria parasite are in circulation in the northeastern of Mali with a seasonal fluctuation of *P. falciparum*. The prevalence of *P. vivax* (9.2%) and *P. ovale* remained stable and was not affected by the season. Two genotypes of *Plasmodium vivax*, Belem and Salvator-1, were identified. The presence of *P*. *vivax* species is related to the presence of Duffy positive populations in that area of Mali. The results suggest that the presence (or absence) of ponds is an overwhelmingly important factor. In contrast to the situation in the more southern (savannah) part of Mali, they also suggest that the significance of seasonal changes is apparent only in communities without ponds.

## Figures and Tables

**Table 1:** Study population repartition by age group in May 2004 (Dry season) and in February 2005 (Cold season).

	0–9 years old						>9 years old					
Sites of study	May 2004		February 2005				May 2004		February 2005			
	*N*	(%)	*N*	(%)	*P*	Total	*N*	(%)	*N*	(%)	*P* (*χ* ^2^ test)	Total

Tidarmène-Ikadewane	47	52.5	132	57.4	0.602	108	61	56.5	98	42.6	0.113	230
Ménaka	203	62.2	44	37	0.001	326	123	37.7	75	63	0.0005	119
Inékar	45	32.8	42	36.2	0.815	113	68	60.2	74	63.8	0.693	116
Anuzegreen	40	36.7	230	66.7	0.0004	109	69	63.3	115	33.3	0.00004	345
Tagalalt	37	33.3	41	28.9	0.762	111	74	66.7	101	71.1	0.473	142
Andéramboukane	44	20.2	33	28.7	0.484	218	174	79.8	82	71.3	0.104	115
N'gouyas	71	61.7	34	28.6	0.001	115	44	38.3	85	71.4	0.0002	119
Tin-abaw	59	51.3	24	20.9	0.012	115	56	48.7	91	79.1	0.0001	115
Tabangout	29	25.7	39	34.2	0.41	113	84	74.3	75	65.8	0.245	114

Total	575	43.3	619	43.7	0.868	1328*	753	56.7	796	56.3	0.866	1415*

*Those numbers are different from the populations we screened because we pulled out the subjects having age as missing value.

In overall, there was not a significant difference among the children less than 9 years of age between May 2004 and February 2005 (*P* = 0.868). In addition, the same observation was also noticed among subjects more than 9 years of age (*P* = 0.866). Subjects between 0 and 9 years of age were comparable during the 2 passages (May 2004 and February 2005) with a *P*  value = 0.868. Similarly, subjects with age greater than 9 years were also comparable between the 2 passages (May 2004 and February 2005) with *P*  value = 0.866.

**Table 2:** Demographic characteristics of study populations.

Demographic characteristics		Ponds	No ponds	*P* value (Fisher test)
2004				
Age group	0–9	414	161	<0.0001
	>9	270	448	
				
Sex	Male	500	209	0.002
	Female	385	234	
				
Derma	Light skin	313	218	<0.0001
	Dark skin	573	225	

2005				
Age group	0–9	476	143	<0.0001
	>9	474	322	
				
Sex	Male	494	238	0.652
	Female	457	233	
				
Derma	Light skin	317	242	<0.0001
	Dark skin	634	229	

This table shows that in 2004 and 2005, there was a significant difference between age groups and the presence of ponds (or absence of ponds); we observed that there were more children in the areas with ponds than areas without ponds (*P* < 0.0001). The same trends were observed with the type of skin (derma); there were more subjects with dark skin in the areas with ponds than areas without ponds during the years 2004 and 2005 (*P* < 0.0001). There were more females in the areas with ponds than areas with ponds than areas without ponds in 2004 (*P* = 0.002); in contrast there was no significant difference between gender and presence of (absence) ponds in 2005 (*P* = 0.625).

**Table 3:** Malaria infection prevalence and morbidity in May 2004 and February 2005.

	May 2004		February 2005		*P* value (Fisher and *χ* ^2^ tests)
Plasmodium specie frequencies	*N *(%)	Total	*N *(%)	Total	
* P. falciparum*	50 (74.13)	64	56 (63.72)	97	0.0124*
* P. malariae*	6 (9.38)	64	21 (22.54)	97	0.0419 *
* P. ovale*	2 (3.13)	64	2 (2.06)	97	0. 9258
* P. vivax*	5 (7.81)	64	10 (10.31)	97	>0.9999
* P. falciparum + P. malariae*	1 (1.56)	64	7 (7.21)	97	0.2060
* P. falciparum + P. vivax + P. ovale*	0 (0)	64	1 (1.03)	97	N/A
Malaria infection and morbidity					
Plasmodic Index	64 (4.95)	1293	100 (7.06)	1415	0.0259 *
Gametocytic Index	6 (0.46)	1293	13 (0.91)	1415	0.2364
Anemia	110 (8.50)	1293	106 (7.49)	1415	0. 3660
Fever	308 (23.82)	1293	310 (21.90)	1415	0. 2557
Splenomegaly	64 (4.94)	1293	41 (2.89)	1415	0. 0077*
Overall malaria symptoms	42 (3.24)	1293	343 (24.24)	1415	<0. 000001 *

*Significant *P*  value.

*Plasmodium falciparum* species decreased significantly in February (*P* = 0.0124); in contrast, there was a marked increased in the frequency *P. malariae* in February (*P* = 0.0419). *P. ovale* and *P. vivax* remained stable between May 2004 and February 2005 (Fisher's exact test, *P* > 0.05). Plasmodic index increased remarkably in February 2005 (*P* = 0.0259); in contrast, the gametocytic index did not vary significantly between March and February but increased in February (*P* = 0.2364). Symptoms such as anemia and fever did not vary significantly between March and February (*P* > 0.05). Splenomegaly decreased significantly in February 2005 (*P* < 0.000001).

**Table 4:** Relationship between the age group and plasmodic index.

Age (in years)	2004			2005			*P* value (*χ* ^2^ test)
	Positives	(%)	Total	Positives	(%)	Total	
0–9	50	(8.69)	575	37	(5.97)	619	0.0753
> 9	14	(1.94)	718	60	(7.53)	796	<0.0001
Total	64	(4.94)	1293	97	(6.85)	1415	0.0441
*P*-value (Fisher's exact test)	<0.0001			0.2890			

The plasmodic index varied significantly from May 2004 to February 2005 in the older people (age greater than 9 years of age) than the group of children between 0 and 9 years of age (Fisher's exact test, *P* = 0.0001). In overall, there was a significant change in plasmodic index between May 2004 and February 2005 (*χ* ^2^ = 4.052; *P* = 0.0441). It was greater in February 2005 with 6.85% versus 4.94% in May 2004.

**Table 5:** Infection prevalence of malaria by site with the presence of pounds and without.

Villages	May 2004		February 2005	
	*N* (%)	Total	*N* (%)	Total
Presence of ponds				
Andéramboukane	7 (3.2)	218	14 (6.1)	230
Ménaka	39 (12)	326	14 (4.1)	345
N'gouyass	1 (0.87)	115	8 (5.6)	142
Tin-Abaw	7 (6.1)	115	3 (2.6)	114
Tagalalt	7 (6.3)	111	41 (34.2)	120

Total	61 (6.89)	885	80 (8.41)	951
	*χ* ^2^=1.49; *P* = 0.221			

Absence of ponds				
Tabangout	0	113	5 (4.3)	115
Tidarmene	1 (0.9)	108	2 (1.7)	115
Anuzegreen	0 (0.0)	108	6 (4.8)	125
Inékar	4 (3.5)	113	7 (6.0)	116

Total	5 (1.13)	442	20 (4.24)	471
	*χ* ^2^ = 8.31; *P* = 0.004			

The prevalence was significantly higher in the villages with ponds than the prevalence obtained in the villages without ponds (*χ* ^2^ = 4.771, *P*  value = 0.0289). Within the study sites where the pounds are located, there was not statistically difference between the 2 follow-ups (*P* = 0.22). In contrast, a significant difference was observed among the village without pounds; malaria infection was higher in February in cold dry season (4.24%) than in May 2004 (1.13%) in the hot dry season (*P* = 0.004). This table suggests the role of the ponds in the transmission of malaria.

**Table 6:** Association between malaria and anemia.

*P. malariae*	Anemia	(%)	Total
Yes	7	(33.3)	21
No	98	(9.7)	1006

Total	105	(10.2)	1027

The prevalence of anemia was 4.6-fold higher in patients with *P. malariae* (33.3%) than in those without (9.7%) malariae species infection (OR = 4.633 95% CI [1.826−11.752]; *P* = 0.003).
